# PANORAMITRA: a novel polyetheretherketone-based perivalvular tissue retractor for use in minimally invasive and conventional cardiac surgery

**DOI:** 10.1007/s11748-026-02284-w

**Published:** 2026-03-24

**Authors:** Yusuke Inaba, Hiroshi Kubota

**Affiliations:** Department of Cardiovascular Surgery, Kyorin University, 6-20-2, Shinkawa, Mitaka, Tokyo, 181-8611 Japan

**Keywords:** Mitral valve repair, Subvalvular exposure, Surgical device, Minimally invasive cardiac surgery, Mitral valve surgery

## Abstract

In degenerative mitral valve surgery, repair typically involves annuloplasty, resection ofdiseased segments, and neochordal implantation for leaflet resuspension. However,subvalvular exposure, particularly of the papillary muscles, remains technicallychallenging under minimally invasive approaches—especially in patients withredundant leaflet tissue.

Here, we describe the clinical application of “PANORAMITRA,” (Senko MedicalInstrument Mfg. Co., Ltd., Tokyo, Japan) a novel perivalvular retractor originally designed and developed by our surgical team, made of medical-gradepolyetheretherketone (PEEK).

The device can be easily deployed through a mini-thoracotomy and precisely positioned within the mitral annulus to facilitate subvalvular visualization. Key words

**Supplementary Information:**

The online version contains supplementary material available at 10.1007/s11748-026-02284-w.

## Introduction

In degenerative mitral valve surgery, repair typically involves annuloplasty, resection of diseased segments, and neochordal implantation for leaflet resuspension.

However, adequate exposure of the subvalvular apparatus—particularly the papillary muscles—remains technically challenging.

This challenge is further amplified in minimally invasive and totally endoscopic mitral valve repair, where limited access, narrow working angles, and restricted instrument maneuverability can compromise procedural accuracy and reproducibility.

Here, we describe the clinical application of “PANORAMITRA” (Senko Medical Instrument Mfg. Co., Ltd., Tokyo, Japan), a novel perivalvular tissue retractor originally designed and developed by our surgical team and manufactured from medical-grade polyetheretherketone (PEEK).

The device can be deployed through a mini-thoracotomy and precisely positioned within the mitral annulus to facilitate subvalvular visualization.

## Technique

At our institution, a right mini-thoracotomy approach is the preferred access for isolated mitral and/or tricuspid valve procedures. Since August 2023, PANORAMITRA has been routinely employed in both mini-thoracotomy and full sternotomy cases. For minimally invasive cardiac surgery, a 3-cm skin incision is created. After exposure of the mitral valve and inspection of valve pathology, the retractor is introduced.

**Fig. 1:**
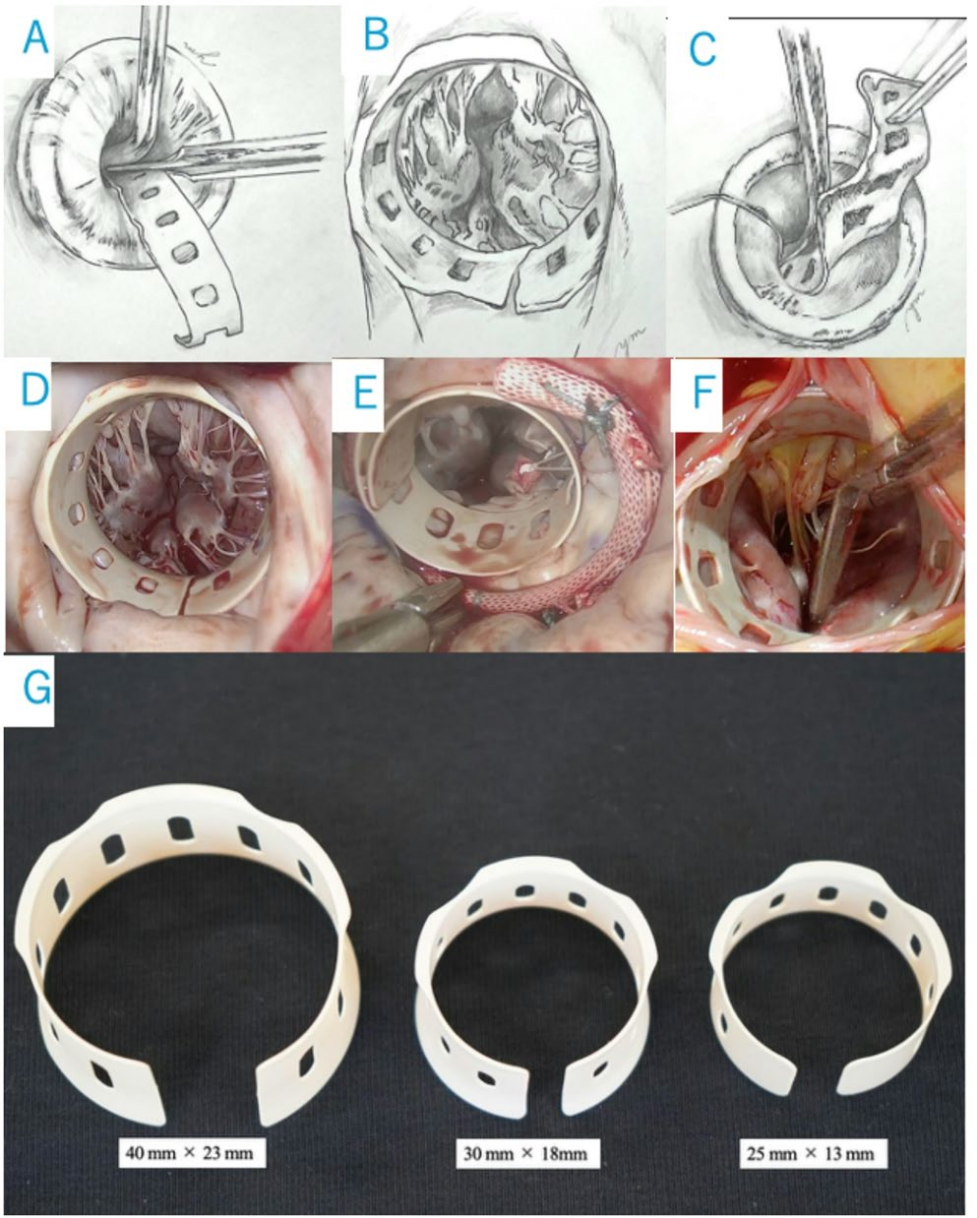
**A** 3-cm thoracic incision is made and the PANORAMITRA device, in its flattened configuration, is introduced through the incision.**B** Once within the left atrium, the device is formed into a controlled spiral using standard minimally invasive instruments, allowing it to traverse the mitral valve and be positioned at the target site.**C** Upon completion of subvalvular manipulation, the device can be easily extracted from the body without tissue injury by gently pulling on one end.** D**PANORAMITRA enables acquisition of a panoramic view of sub-mitral valve tissue organization.** E**Despite implantation of a 30 mm mitral annuloplasty ring, PANORAMITRA enables outstanding exposure of the papillary muscles.** F**Intraoperative view of left ventricular cardiac tumor resection via a transaortic approach (through the aortic valve).** G**Three size variants of PANORAMITRA

Insertion through a 3-cm incision is achieved by introducing PANORAMITRA in a flattened configuration (Fig. 1A), followed by controlled spiraling within the left atrium using standard minimally invasive instruments to allow passage across the mitral valve and deployment into position (Fig. 1B).

Once released within the mitral annulus, PANORAMITRA expands naturally to separate the anterior and posterior leaflets, thereby creating a panoramic view of the subvalvular apparatus (Fig. 1D).

This enhanced exposure facilitates precise and reproducible placement of artificial chordae to the papillary muscles.

After completion of subvalvular manipulation, PANORAMITRA is removed by gently pulling one end of the device (Fig. 1C), allowing atraumatic extraction without interference with previously placed sutures or artificial chordae.

In cases where neochordal implantation is required after annuloplasty, a smaller 25-mm version of the retractor facilitates continued access to the papillary muscles even after ring implantation (Fig. 1E). While PANORAMITRA is particularly beneficial in minimally invasive procedures, it is also effective in sternotomy cases and can be used to expose subaortic structures, such as during left ventricular tumor resection via the aortic valve (Fig. 1F).

The device is available in three configurations—40 mm × 23 mm for enlarged annuli, 30 mm × 18 mm for average annuli and subaortic procedures, and 25 mm × 13 mm for small annuli, including post-annuloplasty access or infective endocarditis cases (Fig. 1G).

## Discussion

Several adjunctive techniques have been proposed to improve subvalvular visualization during mitral valve repair. The Wakka technique described by Tabata et al. [1] uses a ring fashioned from a sterilized paper ruler to separate the chordae and expose the papillary muscles. Although simple and inexpensive, it is disposable and must be manually prepared for each procedure. Erlebach et al. [2] reported the use of aortic valve sizers; however, the rigidity and length of such devices limit their applicability in totally endoscopic procedures with small working incisions. Tudorache and Haverich [3] developed a flexible nickel–titanium retractor that expands within the annulus, but its cost and structural complexity may limit widespread adoption.

From a cost perspective, various adjunctive devices and techniques for subvalvular exposure differ substantially in both upfront expense and practical usability.

Ultra-low-cost approaches, such as manually fashioned leaflet separators, are attractive due to their negligible material cost. However, as discussed above, their performance largely depends on operator technique and may lack consistency across different surgical settings. Other reusable retractors, including valve sizers or flexible metallic leaflet retractors, offer improved structural stability but may be limited by rigidity, shaft length, or insertion complexity, particularly in minimally invasive or totally endoscopic procedures with narrow working spaces.

PANORAMITRA was designed to occupy an intermediate position between these extremes. Although it is not cost-free, it is reusable for up to approximately ten clinical applications. With a list price of approximately USD 650–700, the estimated per-case cost is approximately USD 65–70.

In return for this modest per-procedure cost, PANORAMITRA provides consistent deployability, stable annular fixation, and reproducible panoramic visualization of the subvalvular apparatus across a wide range of surgical approaches. We believe that this balance between cost and reliable clinical performance may be particularly advantageous in complex or minimally invasive mitral valve procedures, where consistent exposure directly contributes to procedural accuracy and reproducibility.

To address the shortcomings and restricted usability of existing devices, particularly in minimally invasive and totally endoscopic mitral valve repair, we designed and developed an original perivalvular tissue retractor based on specific clinical and technical requirements.

PANORAMITRA uniquely addresses these unmet needs through its shaftless configuration, controlled flexibility achieved with medical-grade PEEK, availability in multiple size variants tailored to different surgical scenarios, and slit structures designed to enhance visualization and device stability.

The slit structure was intentionally designed to allow visualization of the mitral leaflets through the device itself during subvalvular manipulation and to provide an anti-slip effect that enhances stability within the mitral annulus.

To prevent material weakening, PANORAMITRA is manufactured from medical-grade PEEK using a machining (milled) process rather than molding. Although specific strengthening techniques applied during manufacturing are proprietary, durability was a primary design requirement.

Mechanical durability testing involving approximately 200 cycles of repeated bending did not result in fracture or permanent deformation.

In clinical use, the device was applied in approximately 20 cases without structural failure; however, surface scratches caused by surgical instruments were observed after repeated use. Based on this experience, disposal after approximately ten clinical uses is recommended to maintain safety and performance.

## Conclusion

PANORAMITRA represents a practical and innovative solution for subvalvular exposure in both minimally invasive and conventional mitral and aortic valve surgery.

By addressing the technical limitations of existing exposure techniques through a design-driven approach, this device improves visualization, enhances stability, and facilitates accurate subvalvular manipulation, thereby supporting procedural reproducibility in minimally invasive cardiac surgery.

## Supplementary Information

Below is the link to the electronic supplementary material.

Supplementary Material 1.
